# Neuromolecular Imaging Shows Temporal Synchrony Patterns between Serotonin and Movement within Neuronal Motor Circuits in the Brain

**DOI:** 10.3390/brainsci3020992

**Published:** 2013-06-21

**Authors:** Patricia A. Broderick

**Affiliations:** 1 Department of Physiology, Pharmacology & Neuroscience, The Sophie Davis School of Biomedical Education, The City College of New York, New York, NY 10031, USA; 2 Department of Biology, The City University of New York Graduate Center, New York, NY 10016, USA; 3 Department of Neurology, New York University Langone Medical Center, NYU Comprehensive Epilepsy Center, New York, NY 10016, USA

**Keywords:** neurotransmitters, behavior, brain, dopamine, serotonin, nigrostriatal pathway, mesocorticolimbic pathway, cocaine, dystonia, spinal cord injuries, central pattern generators, movement disorders, temporal synchrony, atemporal synchrony, drug addiction, psychiatric disorders, neurodegenerative diseases

## Abstract

The present discourse links the electrical and chemical properties of the brain with neurotransmitters and movement behaviors to further elucidate strategies to diagnose and treat brain disease. Neuromolecular imaging (NMI), based on electrochemical principles, is used to detect serotonin in nerve terminals (dorsal and ventral striata) and somatodendrites (ventral tegmentum) of reward/motor mesocorticolimbic and nigrostriatal brain circuits. Neuronal release of serotonin is detected at the same time and in the same animal, freely moving and unrestrained, while open-field behaviors are monitored via infrared photobeams. The purpose is to emphasize the unique ability of NMI and the BRODERICK PROBE^®^ biosensors to empirically image a pattern of temporal synchrony, previously reported, for example, in Aplysia using central pattern generators (CPGs), serotonin and cerebral peptide-2. Temporal synchrony is reviewed within the context of the literature on central pattern generators, neurotransmitters and movement disorders. Specifically, temporal synchrony data are derived from studies on psychostimulant behavior with and without cocaine while at the same time and continuously, serotonin release in motor neurons within basal ganglia, is detected. The results show that *temporal synchrony* between the neurotransmitter, *serotonin and natural movement* occurs when the brain is NOT injured via, e.g., trauma, addictive drugs or psychiatric illness. In striking contrast, in the case of *serotonin and cocaine*-induced psychostimulant behavior, *a different form of synchrony and also asynchrony* can occur. Thus, the known dysfunctional movement behavior produced by cocaine may well be related to the loss of temporal synchrony, the loss of the ability to match serotonin in brain with motor activity. The empirical study of temporal synchrony patterns in humans and animals may be more relevant to the dynamics of motor circuits and movement behaviors than are studies of static parameters currently relied upon within the realms of science and medicine. There are myriad applications for the use of NMI to discover clinically relevant diagnoses and treatments for brain disease involving the motor system.

## 1. Temporal Synchrony between Neurotransmitters and Movement within Motor Circuits

NMI and the BRODERICK PROBE^®^ can be utilized to study movement disorders whether these movement disorders originate in the brain or the spinal cord. This NMI biotechnology actually images neurotransmitters at the same time that movement occurs. Moreover, in dystonic movement injury, e.g., a BRODERICK PROBE^®^ biosensor can be inserted into neurons of muscle and ganglia *in situ* or *in vivo*, enabling real time studies of neurotransmitters within these motor neurons on line while monitoring movement behavior at the same time. Indeed, the brain’s motor neurons, the basal ganglia are the neuroanatomic substrates known to produce sequential movement, adapt reward movement and perform such functions as promoting motor learning and planning. Therefore, such a technological advance as NMI which allows BRODERICK PROBE^®^ biosensors to sense neurochemistry on line with behavior provides potential diagnostic and therapeutic interventions for brain and spinal cord injuries previously unavailable. Presented, are empirical data, performed by NMI, demonstrating a unique temporal synchrony between neurotransmitters and behavior as neurotransmitters are imaged within the basal ganglia nuclei. The most important findings are that (a) *temporal synchrony* between brain neurotransmitter and behavior occurs when endogenous serotonin release in basal ganglia is imaged on line with natural behaviors and (b) psychostimulant-induced behavior, monitored on line while serotonin release is imaged in basal ganglia, produces a different form of synchrony and/or *temporal asynchrony*. Temporal patterns enable a new and dynamic data profile useful clinically and pre-clinically. Although static neurotransmitter levels, currently the standard, are valuable, static parameters become more valuable when empirically studied within the context of movement. Other disorders of basal ganglia, such as athetoid and dystonic disease can be studied with the BRODERICK PROBE^®^. An example of an athetoid, dystonic disease, is Lesch-Nyhan syndrome (LNS). 

LNS is characterized by severe athetoid and dystonic movements, self-mutilation, and repetitive oral stereotypies, similar to fine movement behaviors of licking and grooming observed in rodents which is produced by rewarding drugs, such as psychostimulants. Patients suffering from LNS may be required to have teeth removed to avoid oral stereotypies that cause the patient to devour lips, tongues or fingers. The stereotypies involve dopamine and serotonin [1] and high levels of uric acid [2]. Other athetoid, dystonic diseases and spinal cord injury, drug addiction and psychiatric disorders are discussed within the context of these miniature nano-biosensors that comprise the BRODERICK PROBE^®^ [3,4,5,6,7]; this biosensor is approved by the Institutional Review Board, NYU Tisch Hospital for intraoperative studies in epilepsy patients; studies are published [8].

## 2. The Basal Ganglia

**Figure 1:**
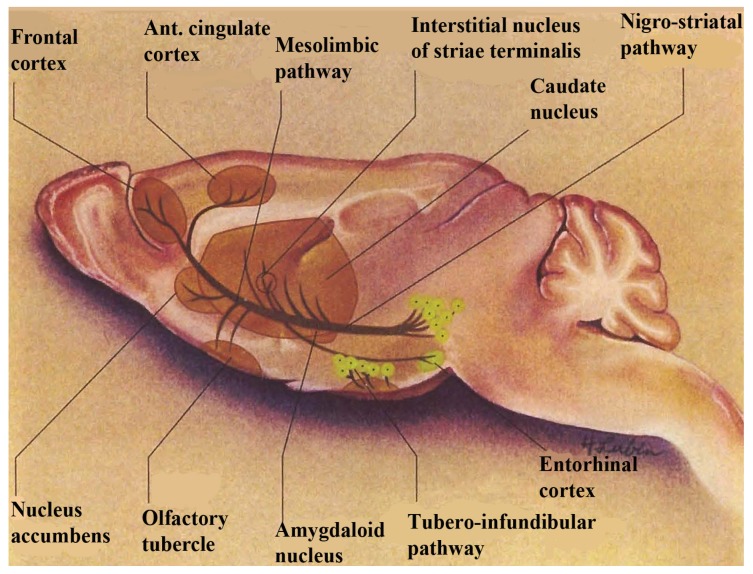
A schematic diagram showing dopamine in nigrostriatal and mesocorticolimbic pathways in basal ganglia. Note: this figure is adapted with permissions from [9,10]. Copyright Humana Press, 2002 and Upjohn Company, 1980.

In order to examine NMI as this biotechnology relates to the experience of temporal synchrony, let us first look at the basal ganglia. The basal ganglia is a complex neuroanatomic substrate targeted by injuries involving movement, cognition and reward, each connected through separate motor and limbic loops within the neuronal ganglia *per se*. The basal ganglia, comprised of basal nuclei are paired subcortical masses or nuclei of gray matter that include the dorsal striatum, (caudate putamen and the globus pallidum) and the ventral striatum. The caudate and putamen are structurally distinct in the human and these structures are joined in lower mammals and are generally considered to function as a unit, called striatum. The terminology, striatum, is derived from the striped nature of this neuroanatomic substrate. Coursing through the striatum and closely related, is the internal capsule which separates striatum from the lenticular nucleus. Also closely associated with the basal ganglia nuclei are small brain-stem nuclei, the substantia nigra, the ventral tegmentum and the subthalamus. The control of voluntary movement is executed by the interaction of the pyramidal, cerebellar, and extrapyramidal systems, which interconnect with each other as well as projecting to the cranial nerve nuclei. The nigrostriatal and mesocorticolimbic pathways are depicted in Figure 1.

Dopamine is a critical neurotransmitter that projects from the substantia nigra to the dorsal striatum (A_9_) with parallel circuitry, the mescorticolimbic (A_10_) neurons emanating from the ventral tegmental area to what is commonly called, “the reward center”, (nucleus accumbens), which is part of the ventral striatum. When we think about the ventral striatum as actually being the “limbic loop” of the motor ganglia which encompasses reward mechanisms, this could possibly mean that stroke victims cannot feel reward in addition to being motor impaired. Therefore, it is important to find medications that treat both the movement and the brain reward disorders. NMI contributed to such a medication for stroke. It was found that Lovenox^®^ a medication for stroke, increased serotonin release in basal ganglia of stroked animals on line, *in vivo*, with increased blood flow. In these studies, NMI simultaneously and selectively imaged neuronal serotonin release in the motor circuit of the murine brain, the dorsal striatum while at the same time, Dual Laser Doppler sensors monitored blood flow [11].

These small gray-matter nuclei, basal nuclei, comprise the basal ganglia. Although these structures lie deep within the forebrain and hindbrain, they are anatomically away from parts of the cortical area and yet, they still have multi-faceted neuronal connections with the cortex. Electrophysiological studies in primates, in addition to movement and cognitive studies in patients with dysfunctional movement, have shown that the basal nuclei operate to assist in movement to (1) determine force and velocity, (2) prepare for movement, (3) develop automaticity, (4) promote sequential movement, (5) inhibit unwanted movement, (6) adapt to novel or reward movement and (7) motor learning and planning [10].

## 3. The Cortico-Basal Ganglia Network

The A_10_ terminal neurons of the mesocorticolimbic circuit is now considered to be the ventral part of the dorsal striatum and this is, as mentioned, the reward circuit, the limbic loop in the basal ganglia. In fact, the reward circuit, is now considered to be embedded within the cortico-basal ganglia network, and is a central component for developing and monitoring motivated behaviors. In the past, the basal ganglia were best known for their relevance to motor functions, based on the neuropathology of movement disorders and the idea that basal ganglia pathways return primarily to motor cortex [12]. Therefore, there has been quite a conceptual leap during the last three decades, with reports showing that the function of the basal ganglia is not a purely motor or sensory-motor worker but the ganglia perform a more complex set of functions that mediate the full range of goal-directed behaviors, including emotions, motivations, and cognitions. The conceptual leap came about from several lines of inquiry and one of these reports, was the demonstration that frontal cortical information passing through the basal ganglia returns to the whole entity of the frontal cortex [13,14].

## 4. NMI in the Dorsal and Ventral Striatum: The Monoamines

**Figure 2:**
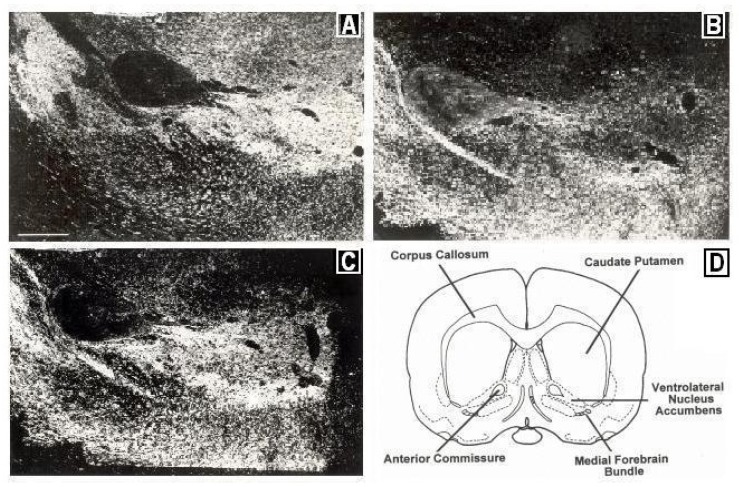
Immunocytographs of dopamine (DA) and serotonin (5-HT) in nucleus accumbens (NAc) (ventrolateral (vl)) of Sprague Dawley laboratory rats. Dark field photomicrographs show the distribution of (**A**) DA neurons, stained with tyrosine hydroxylase; two high density patterns of DA are apparent in the medial and lateral core, (**B**) 5-HT axons in the caudal one-third of NAcc; 5-HT was stained with a sensitive silver intensification procedure, thus axons and terminals are black, (**C**) 5-HT axons in DA neurons in NAcc at the site of the BRODERICK PROBE^®^ laurate biosensor. In (**B**), two low density patterns of 5-HT are apparent in the ventral and ventrolateral NAcc. High density 5-HT is seen in the perimeter around the core. A scale = 500 μm as shown by the horizontal line in the bottom left part of Figure 2A, Direct efferent neurons derive from VTA to vlNAcc. (**D**) Coronal section of NAcc depicting vlNAcc is adapted with permissions from [21]. Copyright Elsevier Limited, 1997.

To look further into NMI, neurotransmitters and brain anatomy, let us look into how the dorsal and ventral striata were distinguished early on by using pharmacology. In pioneering studies by the NOBEL laureate, Dr. Arvid Carlsson, significant amounts of dopamine were found in the basal nucleus, the dorsal striatum, the A_9_ nerve terminal [15]. The somatodendrites for the A_9_ region are called substantia nigra (black body) which Bertler found to contain dopamine as well; the data were reported approximately two years after Carlsson’s finding of dopamine in the dorsal striatum [16]. Monoamine histochemistry soon followed and dopamine was *visualized* in cell bodies and nerve terminals of the A_9_ region by the Falck *et al.* technology [17]. Indeed, the A_9_ region was found to be the largest dopamine containing pathway originating in the subanatomy of substantia nigra, the *pars compacta* [17,18,19,20]. Figure 2 depicts immunocytographs of dopamine (DA) and serotonin (5-HT) in nucleus accumbens (NAc) of Sprague Dawley laboratory animals at the site of the BRODERICK PROBE^®^ biosensor.

Dopamine in the ventral tegmental pathway, cell bodies for A_10_ circuit, adjacent to the nigrostriatal pathway, was reported by Anden *et al.* 1966 [22]. The A_9_ and A_10_ dopamine circuits were further distinguished from each other in terms of psychomotor stimulant behavior. In further studies, the neurotoxin, 6 hydroxydopamine (6-OHDA) was used to lesion the basal nucleus, A_9_ striatum and the result was to eliminate the classical stereotyped repetitive responses of fine movements such as grooming [23]. Then, a psychomotor stimulant was injected into basal nuclei, A_10_, nucleus accumbens and olfactory tubercle and the result was the production of locomotor hyperactivity [24]. There is now an extensive empirical body of evidence pointing to psychostimulant directed dysfunctional movement behaviors due to affected dopamine in basal ganglia. Cocaine was found to increase serotonin as well as dopamine release in nigrostriatal and mesocorticolimbic nerve terminals and somatodendrites in 1992 and 1993 [25,26,27]. In the late nineties, the indoleamine, serotonin was found to be present within the dopamine reward pathway, A_10_, at the site of the BRODERICK PROBE^®^ [9,10,11,12,13,14,15,16,17,18,19,20,21]. 

Dorsal (A_9_) and ventral (A_10_) dopamine pathways have been a major focus of study in this laboratory [25,26,27,28]. Precise distinctions between and within the dorsal and ventral striatal substrates, as delineated by a number of different formulations of the BRODERICK PROBE^®^ are published [3,4,5,6,7]. This is the first laboratory to separately *image endogenous* monoamines, indoleamines and peptide neurotransmitters on line with *natural* movement in the animal subject [9,21]. The focus of the present paper is the indoleamine, serotonin.

## 5. Temporal Synchrony, Central Pattern Generators

Now, let us look into the concept of temporal synchrony, into the phenomenon of rhythmic movement itself, which is often produced by a neuronal network capable of generating a rhythmic pattern of motor activity either in the presence or absence of phasic sensory input from peripheral receptors; this is called a Central Pattern Generator (CPG). Indeed, CPGs have been identified and analyzed in more than 50 rhythmic motor systems and CPGs can generate a variety of motor patterns. A universal characteristic of this wide variety of motor patterns is that they consist of rhythmic and alternating motions of the body or appendages. It is the rhythmicity of these behaviors that make these behaviors appear stereotypic or repetitive. It is the repetitive quality of these behaviors that enables stereotypic behaviors to be controlled automatically. This automaticity or autoactivity means that there may be little or no need for intervention from higher brain centers when the environment remains stable.

The simplest CPGs contain neurons that are able to burst spontaneously. Such endogenous bursters can drive other motor neurons and some motor neurons are themselves, endogenous bursters. Importantly, bursters are common in CPGs that produce continuous rhythmic movement, such as locomotion. But, locomotion is an episodic, rhythmic behavior and thus, further regulation by neurotransmitters, such as dopamine and/or serotonin in motor circuits becomes necessary. Endogenous bursts (cell firing) of neurons involved in locomotion must be regulated by neurotransmitters and neuromodulators, *i.e.*, substances that can alter the cellular properties of neurons involved in CPGs. 

Brief depolarizations occur and lead to maintained depolarizations (plateau potentials) that can last for long periods of time. These maintained depolarizations far outlast the initial depolarization and it is these maintained depolarizations that are necessary for rhythmic movements. The generation of rhythmic motor activity by CPGs can be altered by amines and peptides [29,30], thereby enabling a CPG to generate an even greater variety of repetitive motor patterns. Motor CPGs produce a complex temporal pattern of activation of different groups of motor functions and each pattern can be divided into a number of distinct phases even within a phase. CPGs are also time-dependent [31].

## 6. Serotonin Is a Neuromodulator for CPGs

In fact, serotonin (5-HT) can control the CPG underlying the escape swim response in the mollusc, Tritonia diomedea. Interestingly, the dorsal swim interneurons (DSI’S) are a bilaterally represented set of three 5-HTergic neurons that participate in the generation of the rhythmic swim motor program. Serotonin from these CPG neurons is said to function as both a fast neurotransmitter and as a slower neuromodulator. In its modulatory role, 5-HT enhances the release of neurotransmitter from another CPG neuron, C2 and also increases C2 excitability by decreasing spike frequency adaptation. Serotonin, intrinsic to the CPG, may neuromodulate behavioral sensitization and habituation. Serotonin intrinsic to the DSI enhances synaptic potentials evoked by another neuron in the same circuit [32,33].

In another mollusc, the pteropod Clione limacina, the CPG for swimming is located in the pedal ganglia and formed by three groups of interneurons which are critical for rhythmic activity. The endogenous rhythmic activity of this CPG was enhanced by 5-HT [34]. In the pond snail, Lymnaea stagnalis, 5-HT is the main neurotransmitter in its stereotypic feeding circuit [35]. In the sea slug, Aplysia, the CPG for biting is modulated both intrinsically and extrinsically. Intrinsic modulation has been reported to be mediated by cerebral peptide-2 (CP-2) containing CB1 2 interneurons and is mimicked by application of CP-2. On the other hand, extrinsic modulation of the CPG for stereotypic biting in the sea slug, is mediated by the 5-HT-ergic metacerebral cell (MCC) neurons and this behavior is mimicked by application of 5-HT [36].

In vertebrates, the 5-HT somatodendritic nuclei, the raphe nuclei, comprise the most expansive and complex anatomic and neurochemical system in the Central Nervous System (CNS). Raphe nuclei almost exclusively reside along the midline in the rodent and in the primate. Fewer raphe nuclei reside along the midline [37]. The rostral 5-HT raphe group and caudal linear nucleus sends 5-HT efferents to A_9_ dopaminergic (DA-ergic) basal ganglia motor nuclei and the caudal 5-HT group as well, whereas the interfascicular aspect of the 5-HTergic dorsal raphe projects efferents to A_10_ dopaminergc (DA-ergic) basal ganglia motor nuclei [38].

Electrophysiological studies have shown that the most prominent action of increased 5-HT cell firing in 5-HT somatodendrites, dorsal raphe (DR), is to increase the flexor and extensor burst amplitude of 5-HT cell firing in DR during the act of treadmill locomotion [39]. Further evidence for 5-HT controlling motor output is seen from studies in which 5-HT, directly injected into the motor nucleus of the trigeminal nerve, increased the amplitude of both the tonic electromyogram of the masseter muscle and the externally elicited jaw-closure (masseteric) reflex [40,41,42]. Indeed, Jacobs and Azmitia have proposed that 5-HT’s primary function in CNS neuronal circuitry is to facilitate motor output [38].

Serotonin neurons within 5-HT somatodendrites depolarize with such extraordinary regularity that they exhibit automaticity, *i.e.*, they can act by CPGs and produce plateau potentials. Thus, 5-HT neurons exhibit repetitive discharge characteristics. Increased 5-HT neuronal cell firing in somatodendritic raphe nuclei generally precedes the onset of movement or even increased muscle tone in arousal by several seconds and is maintained during sustained behavior [43]. Importantly, 5-HT cell firing in raphe nuclei is sometimes phase-locked to repetitive behavioral stereotypic responses. The regular firing of 5-HT somatodendrites in raphe nuclei is activated preferentially. This activation is associated with locomotion and chewing, stereotypic behaviors that are stimulated by CPGs [44]. Serotonin intrinsic CPGs have been reported to be responsible for inducing rhythmic motor activity in the spinal cord of the turtle and the lamprey [45,46]. The evidence in the lamprey suggests that 5-HT may have a role in the generation of a family of related undulatory movements including swimming, crawling, and burrowing by using one single CPG.

## 7. Serotonin Is a Neuromodulator for Natural Movement.

NMI with the BRODERICK PROBE^®^ was used to study the continuous empirical relationship between endogenous serotonin release in basal ganglia and natural movement behavior which is directed by basal ganglia *per se*. Specific studies imaging serotonin release in dopaminergic (DA-ergic) motor/reward pathways in (1) the nerve terminals, A_9_, dorsal striatum (DStr), (2) nerve terminals, A_10_, ventral striatum (nucleus accumbens) (NAcc) and (3) somatodendrites (cell bodies), Ventral Tegmental Area (VTA), were performed, *in vivo*. NMI enables empirical studies in each animal subject as its own control. Studies are described in detail in published patents from this laboratory [3,4,5,6,7]; an extensive interpretation and discussion of the neurotransmitter mechanisms involved in motor circuits are reported [9,10,11,12,13,14,15,16,17,18,19,20,21]. Movement behaviors in animals are discussed as open-field behavior with two components emphasized, *i.e.*, ambulations and fine movements [47]. NMI and movement behaviors are synchronized in 5 min intervals. The experimental design for these studies was chosen according to the needs of the open-field behavioral paradigm. The BRODERICK PROBE^®^ images neurotransmitters selectively within a temporal resolution as low as milliseconds.

It is important to describe open-field behavior:

- ambulations (locomotion, walking/running, repeatedly);
- fine movements (stereotypy, behaviors of chewing, licking and grooming);
- rearing (standing movement with front paws extended upward);
- central ambulations (motor behavior taking place in the center of the chamber; central ambulations are believed to be a measure of anxiety, a fear of open places, called agoraphobia/thigmotaxis.

Some important properties of NMI are:

- Each neurotransmitter, imaged selectively via NMI, exhibits a distinct voltage potential at which oxidation or reduction occurs; this results in a recording which has a distinct peak (waveform) for a specific neurotransmitter;
- The concentration of each neurotransmitter within the synaptic environment is directly related to the current as shown by the Cottrell Equation;
- Each neurotransmitter is detected within seconds (semiderivative circuit). Chronoamperometric circuit images within milliseconds;
- NMI is comprised of several formulations and configurations for the BRODERICK PROBE^®^ series of microelectrodes and biosensors.

The following line graphs show *serotonin* release plotted *versus* separate *natural* (*no drug*) open-field behaviors of *Ambulations* (*Locomotion*) and *Fine Movements* (*Chewing*, *Licking*, *Grooming*).

**Figure 3:**
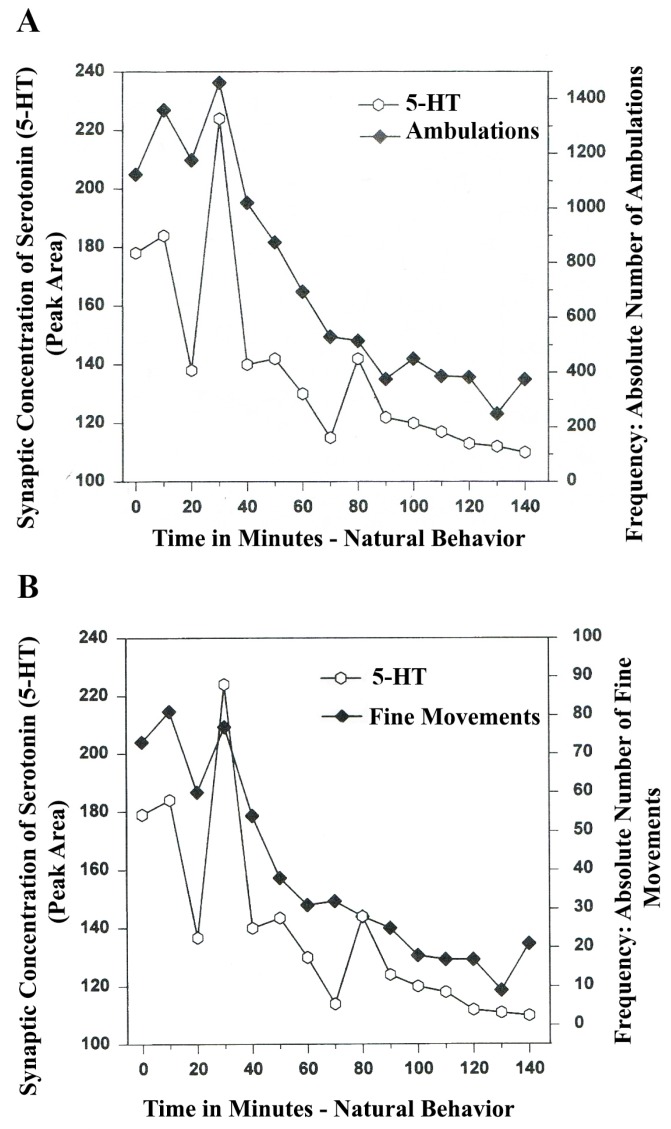
(**A**) *Ambulations*. *Natural neurochemistry and behavior*: line graph depicting endogenous 5-HT release (open circles) at *A_9_ terminals*, *DStr*, detected in real time, while the freely moving, male, Sprague-Dawley laboratory rat is actually behaving, during normal/natural movement (first hour) and subsequent habituation behavior (second hour). (**B**) *Fine movements*. *Natural* (*no drug*) *neurochemistry and behavior*: line graph depicting endogenous 5-HT release (open circles) at *A_9_ terminals*, *DStr*, detected in real time, while the freely moving, male, Sprague-Dawley laboratory rat is actually behaving, during normal/natural movement (first hour) and subsequent habituation behavior (second hour). Note: the figure is adapted with permission from [21]. Copyright Elsevier Limited, 1997.

The results in Figure 3A show that the natural, normal episodic, rhythmic nature of locomotor (ambulatory movement) is neuromodulated by 5-HT within the DA-ergic basal nucleus of the A_9_ terminals, the dorsal striatum (DStr). The data exhibit temporal synchrony between serotonin in dorsal striatum (DStr) and natural open-field behaviors of Ambulations. Figure 3B shows temporal synchrony between serotonin release in the DA-ergic basal nucleus, A_9_, DStr and Fine Movement behaviors.

The terms, synchronous and simultaneous, describe the relationship between serotonin release at distal A_9_ terminal fields *versus* open-field behaviors of ambulations (locomotion) and stereotypic fine movement behavior (chewing, licking, grooming). When serotonin function in motor circuits is endogenous and natural, the co-relationship between the two parameters shows rhythmic regularity.

**Figure 4:**
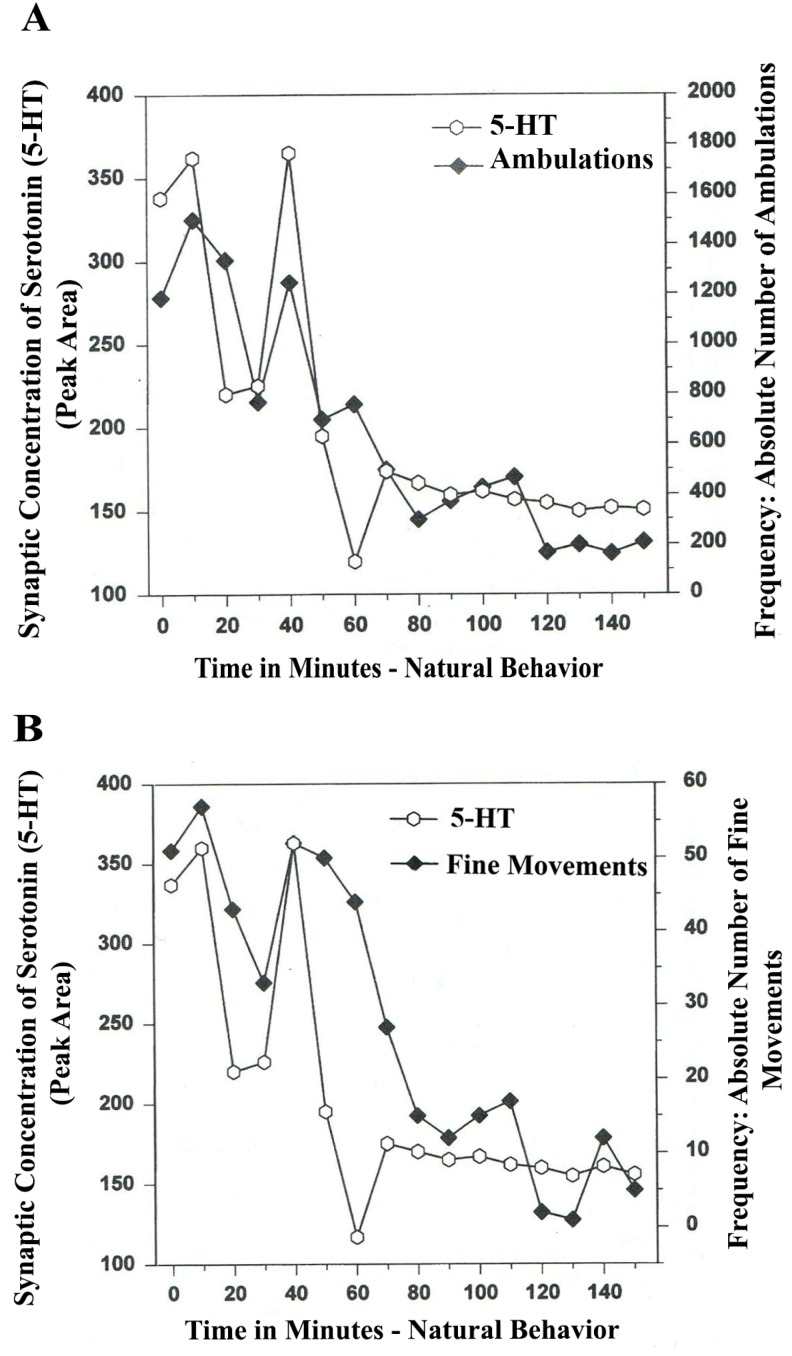
(**A**) *Ambulations*. *Natural neurochemistry and behavior*: line graph depicting endogenous 5-HT release (open circles) at basal nucleus, *A_10_ terminals*, *vlNAcc*, in real time, while the freely moving, male, Sprague-Dawley laboratory rat is actually behaving, during normal/natural movement (first hour) and subsequent habituation behavior (second hour). (**B**) *Fine Movements*. *Natural neurochemistry and behavior*: Line graph depicting endogenous 5-HT release (open circles) at basal nucleus, *A_10_ terminals*, *vlNAcc*, in real time, while the freely moving, male, Sprague-Dawley laboratory rat is actually behaving, during normal/natural movement (first hour) and subsequent habituation behavior (second hour). Note: the figure is adapted with permission from [21]. Copyright Elsevier Limited, 1997. Immunocytographs of dopamine (DA) and serotonin (5-HT) in nucleus accumbens (NAc) (ventrolateral (vl)) of Sprague Dawley laboratory rats.

In Figure 4A,B, serotonin release, imaged in distal A_10_ mesocorticolimbic terminal fields (ventrolateral nucleus accumbens [ventral striatum, (vlNAcc)] with open-field behavior, is shown. 

The results shown in Figure 4A,B show that the natural, normal episodic, rhythmic nature of locomotor (*Ambulatory* Movement) and *Fine Movement* Behaviors are neuromodulated by serotonin (5-HT) within the DA-ergic basal ganglia, the nucleus of the A_10_ terminals, the ventrolateral nucleus accumbens (vlNAcc). Temporal synchrony is exhibited. 

Therefore, the results shown in Figure 3, Figure 4 show that the BRODERICK PROBE^®^ biosensor inserted within nigrostriatal and mesocorticolimbic nerve terminals, (A_9_ and A_10_, respectively) is sensitive to the rhythmic co-relationship between the neurotransmitter, serotonin and movement. In each case, the biosensor is imaging neurotransmitter in the same basal ganglia that directs motor behavior. There is a rhythmic co-relationship between serotonin release at distal A_9_ and distal A_10_ terminal fields and open-field behaviors of ambulations (locomotion) and stereotypic behavior (chewing, licking, grooming). Temporal synchrony occurs in natural neurochemistry and behavior when no drug, injury or trauma is present. 

NMI *in vivo* empirically shows a direct relationship between increased serotonin release at distal presynaptic sites in dopamine motor pathways on line with open-field behaviors of ambulations and fine movements. This is the first data of its kind, demonstrating brain/behavior mechanisms happening with temporally synchronous patterns. These results extend previous voltammetric data, showing that 5-hydroxyindoleacetic acid (5-HIAA), a metabolite for serotonin, is implicated in the awake state of animal subjects [48]. These data, presented herein, further extend and lend an explanatory note to electrophysiological data [37,38,42,43,44] which reports that serotonin directly mediates motor output and maintains steady state function during sustained behavior, for example, as in habituation behavior.

Moreover, what may be of crucial importance, are temporal synchrony patterns in dopamine A_10_ somatodendrites, Ventral Tegmental Area (VTA). What does the temporal communication between serotonin release in VTA and the act of ambulating and/or the fine movement act of chewing look like? Is the increase in serotonin in VTA somatodendrites in tandem before each movement, as electrophysiology studies of *serotonin and movement* have shown? 

**Figure 5:**
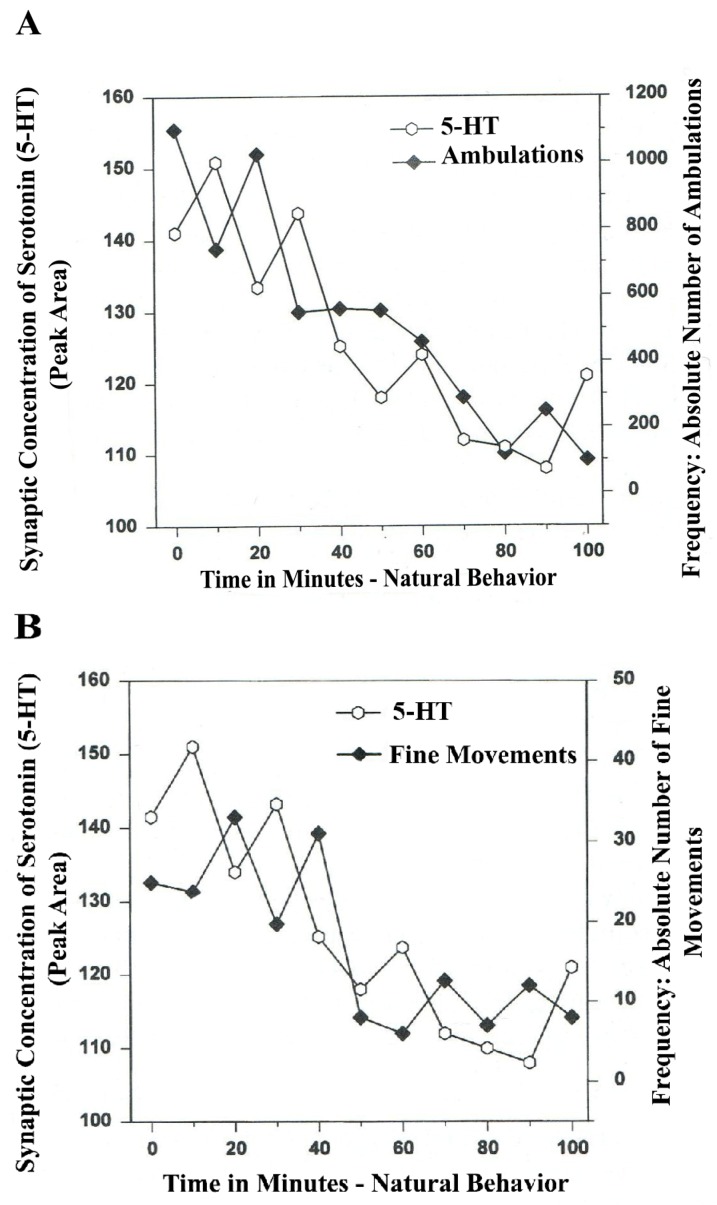
(**A**) *Ambulations*. *Natural neurochemistry and behavior*: Line graph depicting endogenous serotonin (5-HT) release (open circles) at nucleus, *A_10_*, *somotodendrites*, cell bodies, Ventral Tegmentum (*VTA*), in real time, while the freely moving, male, Sprague-Dawley laboratory rat is in the act of behaving, during normal/natural movement (first hour) and subsequent habituation behavior (second hour). (**B**) *Fine Movements*. *Natural neurochemistry and behavior*: Line graph depicting endogenous serotonin (5-HT) release (open circles) at nucleus, *A_10_*, *somotodendrites*, cell bodies, Ventral Tegmentum (*VTA*), in real time, while the freely moving, male, Sprague-Dawley laboratory rat is actually behaving, during normal/natural movement (first hour) and subsequent habituation behavior (second hour). Note: the figure is adapted with permission from [21]. Copyright Elsevier Limited, 1997.

Thus, BRODERICK PROBE^®^ biosensors imaged serotonin release on line with open-field behavior in proximal A_10_ ventral tegmental somatodendrites (VTA). The results are shown in Figure 5.

Figure 5A,B show a natural, rhythmic regularity which is episodic as expected, especially in ambulations (locomotor behavior). The co-relationship between serotonin release in DA-ergic A_10_ somatodendrites and movement behaviors are in juxtaposed temporal synchrony.

Synchronicity describes the co-relationship; patterns are in concert between serotonin release at proximal VTA and open field behaviors of ambulations and fine movements. This additional new finding reveals a synchrony between serotonin release in VTA and open-field behaviors that appears temporally juxtaposed. The comparison of the NMI temporal synchrony patterns in the proximal presynaptic DA somatodendrites *versus* NMI temporal synchrony patterns in neuronal terminal basal nuclei (A_10_) is likely related to the electrophysiology of 5-HT cell firing in somatodendritic cell bodies in 5-HT-ergic raphe nuclei. 

The raphe nuclei project 5-HT efferents to DA-ergic basal ganglia nuclei and act via CPG, stereotypic discharges that can lead to automaticity. When serotonin increases in 5-HT-ergic raphe nuclei, the rise in the firing rate of serotonin neurons generally precedes the onset of movement by several seconds [43]. It is interesting that 5-HT temporal synchrony is juxtaposed in DA-ergic VTA somatodendrites, (the cell bodies) in concert with movement, whereas 5-HT temporal synchrony appears simultaneously with motor behavior within the neuronal terminal basal nucleus (vlNAcc) in the DA-ergic A_10_ circuit. Further work is needed to interpret these novel patterns of brain/behavior mechanisms relating to neurotransmitter function in motor regions of brain. 

## 8. Serotonin Is a Neuromodulator for Psychostimulant-Directed Movement

**Figure 6:**
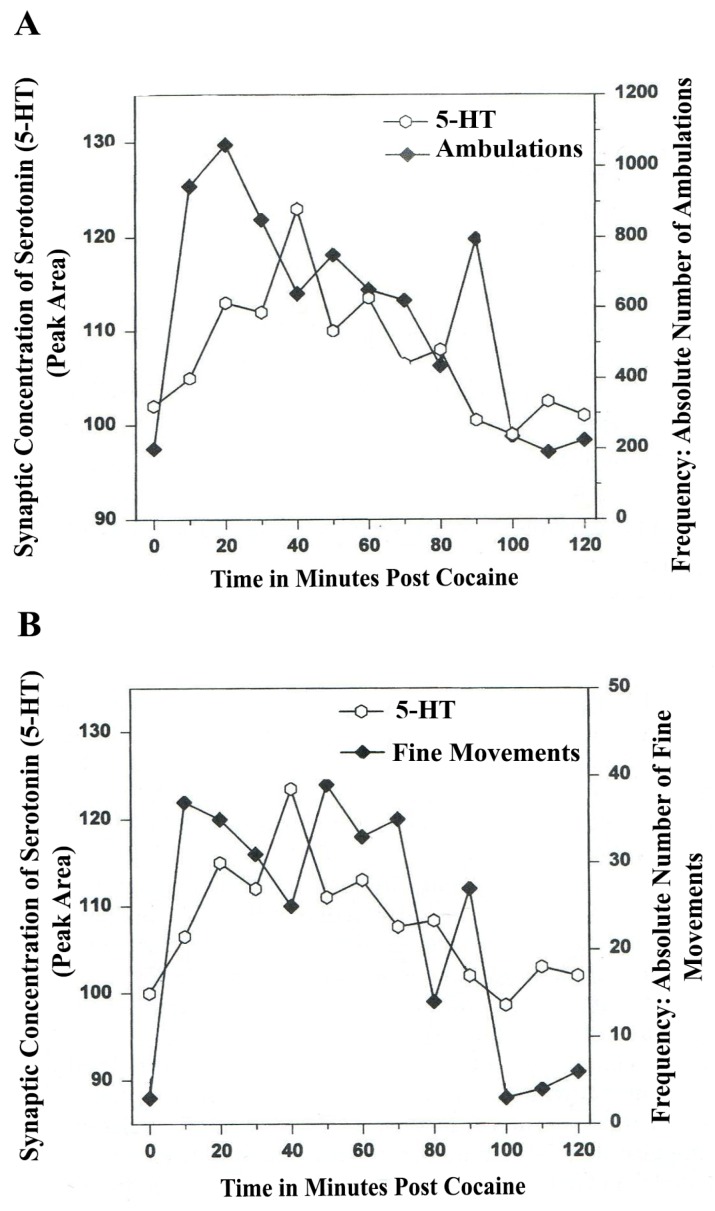
(**A**) *Ambulations*. *Cocaine neurochemistry and behavior*: Line graph depicting endogenous 5-HT release (open circles) at basal nucleus, *A_10_ terminals*, *vlNAcc*, in real time, while the freely moving, male, Sprague-Dawley laboratory rat is actually behaving, during cocaine-induced behavior (cocaine, 2-h study). Baseline is not shown. (**B**) *Fine movements*. *Cocaine neurochemistry and behavior*: Line graph depicting endogenous 5-HT release (open circles) at basal nucleus, *A_10_ terminals*, *vlNAcc*, in real time, while the freely moving, male, Sprague-Dawley laboratory rat is actually behaving, during cocaine-induced behavior (2-h study). Baseline is not shown. Note: the figure is adapted with permission from [21]. Copyright Elsevier Limited, 1997.

**Figure 7:**
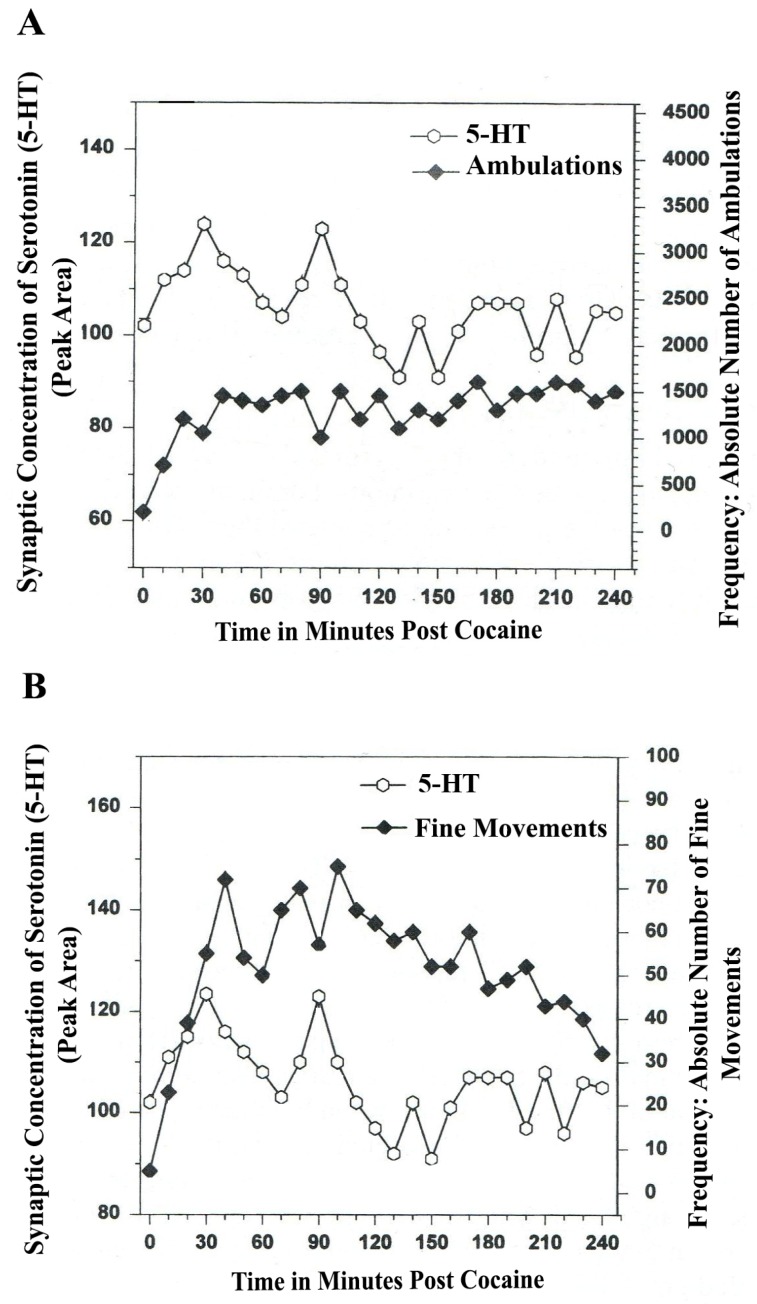
(**A**) Ambulations. Cocaine neurochemistry and behavior: line graph depicting endogenous 5-HT release (open circles) at basal stem nucleus, DA A_10_, somotodendrites, VTA, in real time while the freely moving, male, Sprague-Dawley laboratory rat is actually behaving, during cocaine-induced behavior (cocaine: 4 h). Baseline is not shown. (**B**) Fine Movements. Cocaine neurochemistry and behavior: Line graph depicting endogenous 5-HT release (open circles) at basal stem nucleus, DA A_10_, somotodendrites, VTA, in real time, while the freely moving, male, Sprague-Dawley laboratory rat is actually behaving, during cocaine-induced behavior (cocaine: 4-h). Baseline is not shown. Note: the figure is adapted with permission from [21]. Copyright Elsevier Limited, 1997.

Figure 6, Figure 7 show the disturbing effects of the psychostimulant, cocaine, on temporal synchrony patterns in A_10_ mesocorticolimbic nerve terminals and the disruptive effects of the psychostimulant, cocaine, on A_10_ somatodendrites. These results, derived from psychostimulant-directed movement, are in striking contrast to the results from NMI studies of natural serotonin release and movement behaviors. Whereas endogenous serotonin release in DA-ergic basal ganglia during natural open-field behaviors exhibits temporal synchrony, psychostimulant-directed serotonin release in DA-ergic basal ganglia exhibits different forms of temporal asynchrony.

In Figure 6A and furthermore, in Figure 6B, a form of temporal *asynchrony* is the result of psychostimulant-induced brain/behavior function. It is most intriguing to observe that the psychostimulant effect of cocaine on temporal synchrony in nerve terminal A_10_ basal nucleus remains rhythmic and episodic but not synchronous. The BRODERICK PROBE^®^ biosensor allows such neuroadaptive subtleties to be imaged after a psychostimulant is injected. Such observations as these are critical to future pharmacotherapies for psychostimulant abuse as well as related psychiatric, dystonic movement disorders. It is likely that 5-HT-ergic neurons in raphe nuclei further affect the synchrony of DA neurons in basal ganglia with both types of open-field behaviors studied here. 

The data in Figure 7A,B show that cocaine disrupted the natural, episodic, rhythmic nature of ambulatory and fine movement behaviors, neuromodulated by serotonin (5-HT) within DA-ergic basal stem nucleus, A_10_ somatodendrites. Psychostimulant effects on temporal synchrony appear to occur to a greater degree in somatodendrites as compared with nerve terminal nuclei. 

*In summary*,

- The general and well known upward directional values for the effect of cocaine on serotonin release and movement behaviors in dopamine motor/reward brain circuitry are repeated and confirmed.
- Previous studies have not been able to show these subtle brain alterations during psychostiumulant behavior, nor have these previous studies been able to monitor natural, episodic rhythmic nature of exploratory and habituation behavior on line with the neurotransmitter associated with the specific behavior.
- Serotonin-ergic neuromodulation in A_10_ basal nucleus and A_10_ somatodendrites during natural movement behaviors is disrupted by cocaine.
- There are different forms of asynchrony dependent on whether the neuroanatomic substrate affected is nerve terminal or somatodendrite.
- The studies show that neuronal damage to basal nuclei and brain stem nuclei occurred after the administration of the psychostimulant, cocaine.
- Neuroadaptive responses by serotonin in motor circuits is seen after a single injection of cocaine.
- Neuroadaptation may be a pre-disposition to cocaine neurotoxicity.

NMI data on line with open-field behavior are complementary to the electrophysiological work of Jacobs and Azmitia [37,38,42,43,44]. In the Jacobs and the Azmitia laboratories, the raphe nuclei (somatodendrites for serotonin) and their efferent connections to basal ganglia were the focus; the findings were (1) The rostral serotonin raphe group and caudal linear nucleus sends serotonin efferents to A_9_ basal nuclei motor system and the caudal linear nucleus serotonin group and (2) the interfasciculus aspect of the serotonin dorsal raphe projects efferents to A_10_ mesocorticolimbic basal nuclei, basal ganglia region. 

Thus, NMI enables the imaging of serotonin release in dopaminergic motor circuits in concert with movement behavior. The BRODERICK PROBE^®^ real time data shows the extraordinary regularity, repetitive nature and automaticity of serotonin neurons on line with natural movement behavior in A_9_ and in A_10_ nigrostriatal and mesocorticolimbic dopamine basal ganglia in brain. The data also directly links serotonin with the disruption of natural temporally synchronous behavior known to be caused by psychostimulants and dystonic brain disorders related to psychiatric and mental illness. 

These data link for the first time, the mechanism of neuronal serotonergic central pattern generators (CPGs) in dopaminergic basal nuclei and somatodendrites, specifically with ambulatory and stereotyped movement behaviors. 

## 9. Serotonin Is a Neuromodulator in Spinal Cord Injury

It is known that inflammatory cytokines as well as vasoactive substances such as serotonin are released at the site of spinal cord injury and it has been also shown that serotonin is released in ischemic injury in basal ganglia of animals [11]. *In vitro* work by Saruhashi *et al*., in 2002 [49] shows that the amplitude of action potential necessary for movement in the dorsal column axon of the spinal cord works through different mechanisms, *i.e.*, the serotonin 1_A_ *versus* the 2_A_ receptor. These important studies provide strategies for pharmaceutical therapies in terms of using the serotonin 1_A_ agonist to depress action potentials and assist in acute spinal cord injury. However, in an intact spinal cord injury, signals from the brain are telling the motor neurons to excite or depress causing muscles to contract or expand and since these systems are acting in concert, an *in vivo* model, NMI, becomes more appealing for study.

A recent paper from the Bennett group (2010) [50] studied a virtual “stepping response” to show serotonergic temporal synchrony in a hemisected rodent model of spinal cord injury, *in situ*. This model is valuable especially since many of the neurons that normally coordinate rhythmic movements in mammals are located in the spinal cord [51,52] and spinal neurons require serotonin to get neurons ready to generate movement [53,54]. When the spinal cord is injured, spinal neurons caudal to the injury are serotonin deficient, leaving them “high and dry” in an unexcitable state even though injury did not occur at this caudal site. It is an intriguing finding by the Bennett group that locomotion after spinal cord injury depends on constitutive activity in serotonin receptors [50]. The data, although not *in vivo*, supports our NMI data that ambulations/locomotion are neuromodulated by serotonin. Further evidence for serotoninergic neuromodulation of movement is derived from data showing that locomotion can be regained soon after spinal transection with the exogenous application of drugs that activate the neuromodulatory serotonin, noradrenaline and dopamine receptors *in vivo* and *in vitro* [55,56,57,58], including serotonin 2 and serotonin 7 receptor agonists [59,60,61] or even transplants of serotonin and noradrenaline- producing cells into the spinal cord [62,63]. 

The Bennett group studied a virtual “stepping response: to show serotonergic temporal synchrony, by observing the movement of the hindlimb of the hemisected spinal cord. Our empirical NMI data, *in vivo*, advances the Bennett paper and shows temporal synchrony between serotonin and movement exactly in motor neuroanatomy, the basal ganglia. Our data, shown above, are the first to show that temporal synchrony between serotonin and movement behavior occurs during natural behavior and is rendered dysfunctional during psychostimulant-induced, athetoid and dystonic behaviors. Therefore, in dystonic movement disorders as well as in spinal cord injury in patients and animals, serotonin and rhythmic movement are inseparable. Thus, NMI and the BRODERICK PROBE^®^ provides advances for the diagnosis and treatment of a variety of dystonic movement disorders via the phenomenon of temporal synchrony between neuromodulators and movement directed within motor circuitry.

## 10. Conclusions

Described in this discourse is a dynamic concept of temporal synchrony in natural *versus* drug-induced movement behavior. Temporal synchrony patterns are shown between the neurotransmitter, serotonin, and movement behavior using NMI and the BRODERICK PROBE^®^ microelectrodes and biosensors. Serotonin is imaged in dopamine motor pathways while open-field behavior is monitored with infrared photocell beams in the same animal subject. Temporal synchrony is described within the context of central pattern generators, repetitive discharge in cell firing and automaticity in co-relationship to open-field ambulatory and stereotypic movement behaviors. 

The take-home messages from these studies are new findings [1] *temporal synchrony* between brain neurotransmitter and behavior is exhibited when injury is not present. Brain/behavior mechanisms are rhythmic and synchronous [2] dysfunctional behavior that is asynchronous and arrhythmic comprises *temporal asynchrony*. These temporal brain/behavior patterns using NMI enable the formation of a new and dynamic data profile. Temporal brain/behavior patterns are more relevant to the physician and scientist than are static parameters currently used in research for diagnosis and treatment of brain disease. 

Given the caveat that static levels of neurotransmitters are valuable, static parameters assume more value within the context of movement because dynamic data profiles provide more accurate clinical management. Disorders of basal ganglia, such as athetoid, dystonic disease may be studied with the BRODERICK PROBE^®^. The patient with spinal cord injury will benefit from diagnosis with this biosensor. It is concluded that the healthy brain and spinal cord in humans and animals may very much depend on patterns of brain/behavioral synchronicity. 
